# Sex still matters for the brain and mental health across the lifespan

**DOI:** 10.1186/s13293-025-00710-x

**Published:** 2025-05-13

**Authors:** Adriene M. Beltz, Natalie C. Tronson, Meharvan Singh, Samar Rezq

**Affiliations:** 1 University of Michigan, Michigan, MI 48109 USA; 2 Loyola University Chicago Stritch School of Medicine, Maywood, IL 60153 USA; 3 University of Mississippi Medical Centre, Jackson, MS 39216 USA

*Today*,* on May 13th*,* 2025*,* the Editorial Board of Biology of Sex Differences proudly celebrates Sex Differences in Health Awareness Day. This Editorial*,* authored by members of our own Editorial Board*,* explores the complex relationship between sex differences and the brain*,* with a focus on the vital role of sex differences research in neuroscience and mental health. As part of our Awareness Day campaign*,* this Editorial is co-published alongside a Review article written by additional members of our Editorial Board: “The Role of Sex Differences in Cardiovascular, Metabolic and Immune Functions in Health and Disease: A Review for Sex Differences in Health Awareness Day.” Both articles were commissioned by the Publisher*,* India Sapsed-Foster*,* in recognition of this important day and in support of Sustainable Development Goals 3 and 5—Good Health and Wellbeing and Gender Equality*,* respectively.*

Sex differences are pervasive throughout the brain, with chromosomal differences and exposure to gonadal hormones, such as androgens, during early development in males contributing to divergence of a subset of neural circuits and receptor distribution. In turn, these anatomical and organizational differences contribute to male- and female-specific behavior and physiological responses, which are modified by activational effects of hormone exposure during adolescence and adulthood, and again with hormonal decline during aging. In humans, sex differences in the brain and behavior also interplay with gender – or sex-associated sociocultural roles, norms, and expectations – to contribute to not just *average* differences between men and women, but even to *individual* differences in risk and resilience to mental health and neurological disorders including depression, addiction, and Alzheimer’s disease.

The concept of sex differences, whether anatomical, physiological, cognitive, or behavioral, has been in existence throughout ancient history. In the brain, however, few regions are truly sexually dimorphic (with distinct forms in females and males). In behavior and cognition, sex differences are rarely as simple as “women are smarter than men” or “women are more vulnerable” to a given disorder [1]: Instead, on average women outperform men on some specific aspects of cognition (e.g., verbal memory), and men outperform women on others (e.g., three-dimensional mental rotations). Similarly, male rodents excel at tasks involving spatial information, but this sex difference emerges after puberty, suggesting a hormone-dependent effect, and it also reflects attention to different kinds of information in the environment [2, 3]. Yet, biology is not destiny: Spatial cognition can be modified by experience – with training, the sex difference in many tasks is reduced [4], raising the question of biological versus social (e.g., videogaming experience) contributions to differences, and their intricate interplay across development for unique individuals.

Sex disparities in mental health are also complex. All people can experience depression, addiction, and neurodegenerative disorders including Alzheimer’s disease and Parkinson’s disease. Nevertheless, nearly twice as many females are diagnosed with depression as males (after puberty), whereas males are more likely to develop Parkinson’s disease [5, 6]. Males are also diagnosed with addiction more often than females, although females progress to addiction more rapidly. In each of these examples, gendered factors also play a role: do men seek help for depression at lower rates? Are men exposed to more environmental toxins? Are women exposed to more stress? How do gonadal (or sex) hormones that include estrogens, progestins and androgens impact resilience and susceptibility? And how do changes in hormone levels, both across the lifespan (e.g., puberty, pregnancy, and menopause) and due to exogenous hormones (hormonal contraceptives and hormone therapy) modify neural resilience to disease?

Despite these striking patterns, understanding why and how sex differences in behavior, disease rates, and response to medications has only recently been seriously integrated into biomedical and behavioral research. It was not until 1993 that the United States required that clinical studies include women and minorities to ensure the generalizability of findings from federally-funded research (NIH Revitalization Act). In 2016, 23 years later, NIH enacted a policy requiring sex to be considered as a biological variable (SABV) in preclinical study designs and analyses to ensure equal representation of male and female genetics, cells, and phenotypes in federally-funded basic research. Similar standards have emerged around the world, including the Canadian Institutes of Health Research Sex- and Gender-Based Analysis + policy and the Sex and Gender Equity in Research guidelines, adopted by many European-based academic publishers [7].

With these structural changes, there has been progress! Sex-related diagnostic features, risk factors, and treatments have been established in a multitude of neurological and mental health conditions, providing critical insights into early detection and effective intervention for all. When research findings represent the sexes and generalize across them – even with qualifications and caveats – then harmful conditions can be fully understood in ways that benefit everyone. Yet, there is much work to do! Multilevel challenges continue to plague sex differences research, ranging from political divisiveness to paltry funding for the study of female-predominant conditions to the inappropriate consideration of sex differences in analyses even when sex is incorporated into study designs [8, 9]. Thus, we take the occasion of Sex Differences in Health Awareness Day 2025 to reify the necessity of research on sex-related aspects of neuroscience and mental health across the lifespan.

Because the extant data – particularly in basic neuroscience – is primarily derived from males, sex differences research is especially crucial for understanding factors that pertain to females. Among the critical factors thought to underlie sex differences in anatomy, physiology, cognition, and behavior are gonadal hormones. The many functions of gonadal hormones include contributions to sex differences in development and disease vulnerability as well as responses to pharmacological interventions. To illustrate the elegance of neuroendocrine systems for neuroscience and mental health, we focus on the ways in which scientific, clinical, and public understanding of hormonal contraceptives (HCs) in reproductive-age females and then Alzheimer’s disease (AD), especially surrounding the menopause transition, have recently been transformed when scientists applied a sex difference lens to empirical approaches, and we highlight research opportunities for the near future in these domains crucial for wellbeing across the lifespan. We draw on studies across multiple species, including humans, that illustrate how gonadal hormones influence brain physiology and development as well as subsequent cognition and mental health.

Pubertal development is largely sex-specific, with primary and secondary sexual characteristics being directly linked to gonadal hormone production and function. Sex differences in many mental health conditions also emerge during puberty, including the female preponderance of depression and the male prevalence of conduct disorders and subsequent addiction [1]. After physical maturation, many females use HCs to treat a slew of possible reproductive health problems and to facilitate responsible reproduction and personal autonomy. With about 400 million users, spanning an age range from adolescence to menopause, HCs are one of the most widely used classes of drugs worldwide [10].

HCs are composed of synthetic hormones. HCs include a progestin component, and some also contain an estrogen, which act as negative feedback on the hypothalamic-pituitary-gonadal (HPG) axis to decrease endogenous progesterone and estradiol, suppressing ovulation, and/or modifying the intrauterine environment. By both acting as agonists at progestin and estrogen receptors, and by suppressing circulating gonadal hormones, HCs also modulate neural function in interesting – and informative – ways. For example, HCs come in many formulations, and some formulations also act at androgenic receptors either as an agonist or exerting an anti-androgenic effect. Thus, by studying the effects of HCs on the brain, on cognition, and on resilience and vulnerability to mental health and neurological disorders, the impact of hormonal states on the brain and contributions of HCs to health and disease can be revealed.

For example, HCs have been linked to increased risk for depression diagnoses and treatment in several population-wide studies, with risk elevated for teen users and those with hormonal sensitivities [11]. Nevertheless, this risk affects a relatively small proportion of individuals, with others experiencing beneficial effects on mood. There is also some indication that users who tolerate HCs well see mental health benefits with long-term use [12]. Thus, among the most pressing issues for future research concern individual differences – and even personalization. The goal is to determine – in advance – who is at risk for negative mental health side effects and then leverage information about the vast heterogeneity in HC formulations to try to match users to an option that will achieve their reproductive health aims, with minimal impacts on their unique mental health.

Experimental work from both HC users and in rodent models provides some insights into how modifying gonadal hormones with HCs may contribute to risk or resilience to depression. HCs reliably suppress the hypothalamic-pituitary-adrenal axis response to acute stress in humans, and in rodent models of HC exposure [13]. This is not surprising – the HPG and HPA axes are closely intertwined. Given the central role of dysregulated HPA axis in the etiology of depression, the hormonal and molecular interactions of HCs with stress responsivity are likely key mechanisms by which HCs modify depression risk [14]. 

Identifying how HCs modify depression risk has crucial implications for understanding sex differences. First, gonadal hormone interactions with stress also likely contribute to individual differences in resilience and susceptibility to depression, including prior experience and genetic factors. Second, identifying how exogenous hormones modify risk will provide new avenues for identifying mechanisms that contribute to disparities in depression (as well as insights into other hormone-related mood disorders, including premenstrual dysphoric disorder and postpartum depression).

There are also striking sex differences in cognitive decline and AD [15]. Gonadal hormones play an important role in shaping brain health with advancing age across species. For example, estrogens are highly neuroprotective throughout the lifespan, and estrogen loss surrounding menopause seems to contribute to increased prevalence of aging related cognitive decline in females. This also extends to AD. More than twice as many females as males are diagnosed with AD, and females show more rapid decline with the diagnosis after menopause. Again, estrogens seemingly confer a protective effect, both prior to menopause and with supplementation after menopause.

Sex differences in AD are more complex than a seemingly protective role of available estrogens, though. For example, prior to menopause, females outperform age-matched males in some aspects of cognition, particularly verbal memory and executive function [16], providing some resilience to cognitive impairments during aging. There are also sex differences in genetic associations, such that females have stronger associations between apolipoprotein E (APOE-ε4), a major genetic risk factor for late-onset AD, and cognitive decline than do males [17]. Similarly, females show more neuropathological features of AD, such as amyloid plaques, tau deposition, and neurofibrillary tangles (NFT) [18, 19]. It is somewhat surprising, therefore, that new pharmacological treatments for AD that decrease amyloid burden are more effective for alleviating cognitive decline in males than females [20]. Importantly, these findings clearly demonstrate that sex differences across multiple dimensions contribute to rates, resilience, progression, and treatment of AD. As yet, we can only begin to guess at some of these sex- and gender-specific contributors. This is a pressing area for future research.

In this editorial and in honor of Sex Differences in Health Awareness Day 2025, we have highlighted key advances that have helped provide mechanistic foundations for the biology of sex differences focusing on HCs and AD, noting that science has come a long way, but more progress is required. HC and AD research are not only valuable in their own right, but they also serve as illustrations of how hormones and other sex-related factors influence neurological and mental health conditions. Basic and translational research aiming to further unravel the mystery of sex differences via a precise delineation of gonadal hormone contributions to what goes right in healthy development is sorely needed. Importantly, sex differences research in humans is intertwined with sociocultural norms and expectations around gender-appropriate behaviors and roles. Only when there is a full understanding of convergences and divergences across sex and intersections with gender, can inferences be made about how best to treat disorders, to ensure an optimal healthspan for all.
